# Interleukin-18, Functional IL-18 Receptor and IL-18 Binding Protein Expression in Active and Latent Tuberculosis

**DOI:** 10.3390/pathogens9060451

**Published:** 2020-06-08

**Authors:** Sebastian Wawrocki, Grzegorz Kielnierowski, Wieslawa Rudnicka, Michal Seweryn, Magdalena Druszczynska

**Affiliations:** 1 Department of Immunology and Infectious Biology, Institute of Microbiology, Biotechnology and Immunology, Faculty of Biology and Environmental Protection, University of Lodz, Banacha 12/16, 90-237 Lodz, Poland; sebastian.wawrocki@biol.uni.lodz.pl (S.W.); wieslawa.rudnicka@biol.uni.lodz.pl (W.R.); 2 Regional Specialized Hospital of Tuberculosis, Lung Diseases, and Rehabilitation, Szpitalna 5, 95-080 Tuszyn, Poland; kielnier@o2.pl; 3 Center for Medical Genomics OMICRON, Jagiellonian University, Medical College, Swietej Anny 12, 31-008 Cracow, Poland; michal.seweryn@wmii.uni.lodz.pl

**Keywords:** tuberculosis, IL-18, IL-18BP, IL-18R, gene expression

## Abstract

A thorough understanding of the processes modulating the innate and acquired immune response to *Mycobacterium tuberculosis* (*M.tb*) infection in the context of gene expression is still a scientific and diagnostic problem. The study was aimed to assess IL-18, IL-18 binding protein (IL-18BP), IL-18R, IFN-γ, and IL-37 mRNA expression in patients with active tuberculosis (ATB) and healthy volunteers with latent *M.tb*-infection (LTB) or *M.tb*-uninfected healthy controls (Control). The relative mRNA expression was assessed in the buffy coat blood fraction using the qPCR method. In total, 97 BCG-vaccinated Polish adults were enrolled in the study. The relative expression of IL-18 and IL-18BP mRNA was significantly elevated in the ATB and LTB groups. In ATB, but not LTB individuals, the overexpression of IL-18 and IL-18BP, as well as a significant increase in IFN-γ mRNA expression, might be considered as a manifestation of active tuberculosis disease. No statistically significant differences were observed in the IL-37 mRNA expression among the studied groups. Particularly noteworthy is the outstanding reduction in the relative expression of IL-18R mRNA in the LTB group as compared to the ATB and Control group. Reduced expression of IL-18R in LTB group may, at least partially, prevent the development of a pathological inflammatory reaction and promote the maintenance of homeostatic conditions between host immunity and *M.tb.*

## 1. Introduction

Tuberculosis (TB) is the leading global cause of death from an infectious agent, *Mycobacterium tuberculosis* (*M.tb*). TB affects about 10-million people in the world and is a cause of two-million deaths annually, according to the estimates of the World Health Organization [1]. One-third of the world population carries an asymptomatic *M.tb* infection. These individuals have developed an efficient immune response that allows them to block the metabolic activity of the pathogen, but it does not provide its eradication. People with a latent TB infection (LTB) represent a reservoir of potential progress to disease, because about 5–10% of them will develop active TB disease, if not treated.

The antigen-specific, as well as non-specific, response of the immune cells to *M.tb* infection is modulated by mRNA expression, which results in the production of cytokines and other proteins activating numerous cell populations. Among these, interleukin (IL)-18plays an important role—it induces NK cell cytotoxic activity and promotes the development of Th1 cell response. This mechanism is associated with the production of interferon (IFN)-γ, which is a key element in anti-mycobacterial protection. IL-18 was first described in 1989 as an “IFN-γ inducing factor” [2,3,4]. Similarly to IL-1β, IL-18 is constantly synthesized as an inactive precursor, and the cysteine protease (caspase-1) is involved in its maturation. In a mouse model, increased susceptibility to infection with *M.tb* was found in animals that were not able to produce IL-18 [5]. Moreover, IL-18 increases the expression of adhesion molecules, the synthesis of enzyme nitric oxide synthase, and the production of chemokines. In addition to inducing a T helper (h) type 1 (Th1) cellular response, IL-18, together with IL-2, leads to a Th2 type cell response and the production of IL-4 and IL-13 [6]. In humans, the gene encoding IL-18 is located on chromosome 11 at position 11q22.2–q22.3 and it consists of six exons. There are several common polymorphisms in the promoter region of IL-18 than affect the transcription factor binding sites and, in turn, might be expression quantitative trait loci (eQTLs) for IL-18. Therefore, these genetic variants may predispose to TB by affecting the expression of the cytokine itself, followed by the development of the IFN-γ-mediated Th1 response [7].

The IL-18 levels are also regulated by soluble IL-18 binding protein (IL-18BP), which is a natural inhibitor of IL-18. Under physiological conditions, the concentration of plasma IL-18BP is ~20 times higher than that of IL-18, which prevents IL-18 from binding to its cellular receptor. The gene coding for IL-18BP is located on chromosome 11 at position 11q13.4. The mRNA promoter region contains two response elements (RE), regulatory sequences sensitive to IFN-γ attachment, which results in increased gene expression and protein production [8,9].

Cell activation by IL-18 occurs via the IL-18R receptor, which belongs to the IL-1 family, the members of which show structural and functional similarity. IL-18R is expressed on many cells, such as macrophages, NK cells, neutrophils, epithelial cells, and smooth muscle. The IL-18R receptor is a heterodimer that is composed of two polypeptide chains: IL-18Rα and IL-18Rβ. The IL-18Rα chain is responsible for ligand binding. However, it binds to IL-18 with low affinity. On the other hand, the IL-18Rβ chain functions as a co-receptor, which enhances the strength with which the receptor binds IL-18 and transmits the signal to the inside of the cell [10,11,12]. The genes encoding the receptor are located on chromosome 2 at position 2q12.1 There are known genetic variants in the regulatory regions of IL-18R associated genes, which may affect its and functionality [13].

A new member of the IL-1 family, IL-37, has gained increasing attention in recent years. IL-37, to which IL-18BP also has a high affinity, was found to be an important regulator of inflammation [14,15]. IL-37 is also able to bind to IL-18Rα, but with much lower affinity than IL-18 [16]. IL-18BP and IL-37 act to reduce the production of inflammatory cytokines; however, the anti-inflammatory properties of IL-37 depend on the concentration of IL-18 binding protein. The human IL-37 gene cluster is located on chromosome 2 at position 2q14.1 [17].

IFN-γ is a cytokine that is known to be crucial in regulating the immune response to *M.tb* infection. This cytokine is mainly produced by activated CD4 (+) Th1 T lymphocytes, and it also has a key role in inducing nitric oxide (NO)-dependent apoptosis of mycobacteria infected macrophages. IFN-γ was shown to increase the expression of major histocompatibility complex (MHC) class I and II surface molecules and promote differentiation towards Th1 response. Deleterious mutations in the gene encoding the IFN-γ receptor predispose to the acute course of mycobacterial infection [3,18].The human immune interferon gene is located on chromosome 12at position 12q15 [19]. The expression levels of these cytokines, as well as their mutual relations in sera, as well as in cultures stimulated, were found to be informative of *M.tb* infection status [20,21]. This work aims to assess the level of expression of IL-18, IL-18BP, IL-18R, as well as IFN-γand IL-37 genes in patients with active pulmonary tuberculosis (ATB), healthy individuals with latent *M.tb* infection (LTB), and healthy uninfected controls (Control).

## 2. Materials and Methods

### 2.1. Study Subjects

A study group consisted of 97 adults that were vaccinated with *M. bovis* BCG in childhood, including 51 patients with active pulmonary ATB (40 males, 11 females) aged 23–80 years, hospitalized and diagnosed in the Regional Center Hospital for Tuberculosis, Lung Diseases, and Rehabilitation in Lodz, Poland, 24 healthy volunteers, eight males, 16 females, with LTB (25–65 years old), and 22 healthy uninfected Controls (five males, 17 females), aged 18–66 years (Table 1). Active pulmonary TB was diagnosed by chest radiography and standard clinical examination—by Ziehl–Neelsen staining of sputum smears and *M.tb* culture as a gold standard. The Ethics Committee of the University in Lodz, Poland approved the study (ethical approval number 17/KBBN-UŁ/II/2016; date 2016/11/10). Informed consent to use blood for research purposes was signed by all participants.

### 2.2. RNA Isolation

RNA isolation from the buffy coat obtained after centrifugation (150 g, 4 °C, 10 min.) was performed by the use of a commercial QIAamp^®^ RNA Blood Mini set. Genetic material was isolated from 3.5 mL of peripheral blood obtained from all volunteers using EDTA tubes and the BD Vacutainer^®^ Blood Collection system. The isolation process was fully compliant with the manufacturer’s guidelines. The isolation process was extended by an additional purification step using the RNase-Free DNase Set to obtain the purest product free of any genomic DNA. All of the procedures were carried out within no more than 2 h from the collection of a blood sample. Part of extracted RNA was used to visualize a product and obtain cDNA immediately after the isolation process; the rest of the genetic material was stored at −80 °C until analyzed.

### 2.3. Spectrophotometric Evaluation of Isolated RNA and Gel Visualization

At the end of the isolation procedure, 1.5 μL of each sample was pipetted into a sterile Eppendorf tube, which was then placed in an ice block, in order to assess the quality and quantity of RNA obtained. An additional water-containing blank was prepared to calibrate the device (NanoDrop), which was used for RNA elution in the final isolation step. For visualization of RNA, 1.2% agarose gel was prepared based on TAE buffer. Agarose gel was enriched after cooling with 10 μL of 5 mg/mL ethidium bromide. Each sample of RNA in a volume of 3.5 μL was heated to 70 °C in a water bath for 1 min. and then cooled on ice for another minute. An equal amount of loading buffer was then added to each of the samples, and the mixture was then loaded to the gel. The GeneRuler Plus DNA Ladder size 100 bp from ThermoFisher was used as the size standard. Electrophoresis was carried out at 90 V for 60 min. Subsequently, to visualize the obtained product, the gel was transferred to a Gel-Doc 2000 apparatus that was connected to a computer with Quantity One software. The analysis of the obtained gel allowed a clear distinction between the two isolated RNA fractions: 18S RNA and 28S RNA.

### 2.4. Reverse Transcription

cDNA was synthesized according to the manufacturer’s instructions of the iScript™ cDNA Synthesis Kit (Bio-Rad). In the first stage of the reverse transcription reaction, 1 μg matrix RNA previously tested for quality and integrity was transferred from an isolated sample to 0.2 μL Eppendorf tubes, followed by the addition of reagents that are necessary for the cDNA synthesis process according to the manufacturer’s proportions, which results in a mixture with a final volume of 20 μL. The reverse transcription reaction was carried out in a Biometra UNO II thermal cycler under conditions following the manufacturer’s guidelines. The resulting cDNA was stored at −20 °C until analyzed.

### 2.5. qPCR Reaction

qPCR was performed in a CFX96 Real-Time PCR Detection System (Bio-Rad). The reaction mixture (10 μL) contained 5 μL of iTaq universal SYBR Green Supermix, 0.5 μL of each primer (10 μM), 1 μL of cDNA, and 3 μL of nuclease-free water. Amplifications were performed using the following cycling profile: an initial activation step (95 °C for 3 min.) followed by 40 cycles of denaturation at 95 °C for 10 s, annealing at a temperature appropriate for selected starters (Table 2) for 10 s, and extension at 72 °C for 20 s. For melting curve analysis, a dissociation step cycle (60 °C for 5 s, and then 0.5 °C for 5 s until 95 °C) was added. All of the qRT-PCR experiments were performed in three technical replicas.

Analysis of gene expression was done through a comparative method (ΔΔCt) in order to determine the relative level of expression of selected mRNAs. This method is based on calculating the differences in the level of expression of the test gene and the reference gene. The calculations use the threshold cycle (Ct) values of the qPCR reaction. Ct values were determined for both the test and reference genes in both the test and control samples, for which the differences between the individual Ct values (ΔCt) were then calculated.

### 2.6. Statistical Analysis

The expression of genes between the study groups was compared using Kruskal–Wallis’ non-parametric diagnostic test. A *p*-value < 0.05 was considered to be statistically significant. Statistical analyses were done using MedCalc (MedCalc Software, Ostend, Belgium) and GraphPad Prism 8 (GraphPad Software, La Jolla, CA, USA) software.

## 3. Results

### 3.1. Selection of Reference Genes

**Figure 1:**
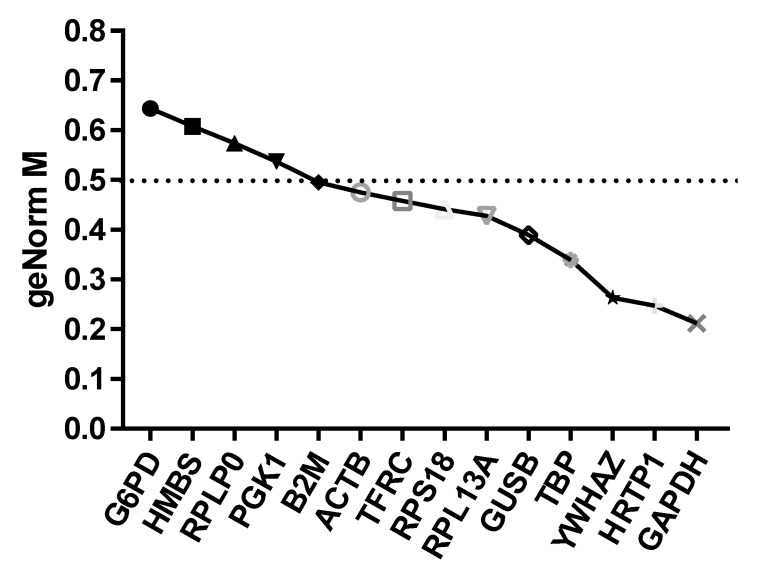
Chart showing the most (lowest M value) and least stable (highest M value) reference genes indicated by the program GeNorm.

We had chosen to use the Reference Gene qPCR Panel BioRad with primers being designed and coated on the plate in lyophilized form for selected reference genes adequate for the analyzed material obtained from volunteers [26]. The housekeeping genes were: ACTB, RPL13A, B2M, RPLP0, G6PD, RPS18, GAPDH, TBP, GUSB, TFRC, HMBS, YWHAZ, HPRT1, PGK1, and IPO8. Additionally, the panel contained several internal controls ensuring the most reliable results. After the RT-PCR reaction, the analysis of the melting curves for the obtained amplicons was carried out in order to determine the quality of the reaction. In the next stage, the Ct values that were detected for individual genes were used for further analyses, which were carried out using the BioRad program (CFX Manager™ Software), and the GeNorm program. As a consequence of the analysis, we decided to use the following reference genes: GADPH, HPRT1, and TBP. These genes had the lowest M value corresponding to the most stable gene expression in the sample tested. The analysis of Ct values using the geNorm program allowed for further refinement of this gene list and, in turn, only HRTP1 and GAPDH were used as the reference panel, as shown in Figure 1.

**Figure 2:**
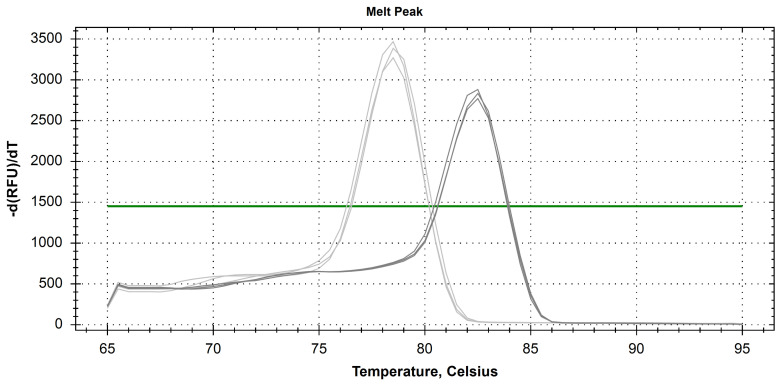
Melt peak analysis of reference genes HPRT1 (light grey) and GAPDH (dark grey).

Melt peak analysis demonstrated a single homogenous peak for all primer sets, including selected reference genes (Figure 2).

### 3.2. IL-18, IL-18BP, IL-18R, IFN-γ, and IL-37 Gene Expression in the Studied Groups

**Figure 3:**
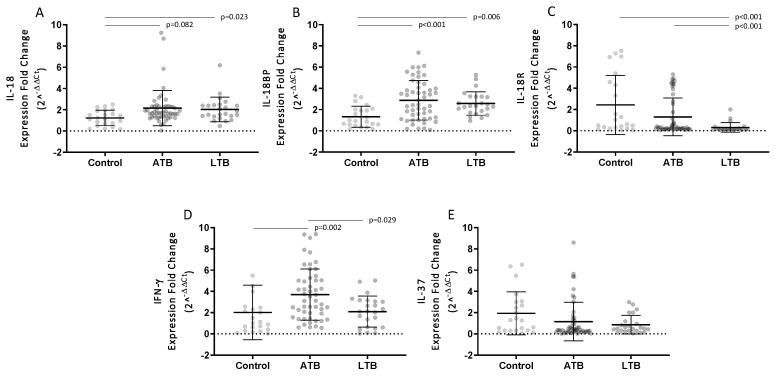
Relative expression of IL-18, IL-18BP, IL-18R, IFN-γ, and IL-37 mRNA in studied groups. Dot plot with mean (horizontal line), and standard deviation (whiskers), IL-18 (**A**), IL-18BP (**B**), IL-18R (**C**), IFN-γ (**D**), IL-37 (**E**) in the groups of healthy volunteers (Control), patients with active tuberculosis (ATB), and latently infected individuals (LTB).

A significantly higher relative level of IL-18 mRNA expression was observed in LTB individuals as compared to healthy controls without *M.tb* infection (*p* = 0.023). A similar increase in the relative IL-18 expression level was observed among ATB patients; however, the difference in values for the group ATB and group Control group was not significant (*p* = 0.082) (Figure 3A).

The relative level of IL-18BP mRNA expression was significantly higher in ATB patients (*p* < 0.001) and healthy LTB individuals (*p* = 0.006) than in Control group volunteers (Figure 3B).

There were major significant differences between the three groups in the IL-18R mRNA expression. In the LTB groups, the relative level of IL-18R mRNA was much lower than in the ATB patients (*p* < 0.001) and individuals from the Control group (*p* < 0.001) (Figure 3C).

The level of relative expression of IFN-γ mRNA was significantly higher in active TB patients (*p* = 0.002) and LTB individuals (*p* = 0.029) than in the Control group (Figure 3D).

No statistically significant differences were observed in the levels of relative expression of IL-37 mRNA among the studied groups (Figure 3E). 

## 4. Discussion

A unique feature of *M.tb* is the ability to persist in the host for a long time, despite functioning mechanisms of acquired immunity. In turn, approximately 2.3 billion individuals have latent tuberculosis infection without evidence of the clinical manifestation of active TB. It is hypothesized that active TB is usually caused by the reactivation of endogenous infection and untreated LTB is a major source of new active TB infections and transmission. The risk for severe active tuberculosis reactivation is increased several times among the immunocompromised individuals, diabetics, organ transplantation recipients, patients with hematologic malignancies, or HIV-infected subjects. Several treatment regimens of LTB are recommended, between isoniazid monotherapy for six months, rifampicin plus isoniazid for three months or rifapentine plus isoniazid for three months [27]. Currently, interferon-gamma release assays (IGRA) are used to test LTB based on the production of IFN-γ by Th1 cells responding to specific *M.tb* antigens, although the IGRA’s accuracy in immunocompromised individuals is still limited. Moreover, the definition of active tuberculosis infection might not be accurate using IFN-γ responses to *M.tb* antigens even in combination with tuberculin skin test [28]. At the same time, neither the IGRA test nor the tuberculin skin test can distinguish between latent and active TB infection. Strategies for rapid differentiation of patients with active TB and people with LTB and prevention of tuberculosis reactivation in LTB individuals are urgently needed. 

To meet such needs, we compared the expression of genes of the IL-18 pathway, functional receptor of this cytokine IL-18R, and IFN-γ, as well as the expression of IL-18BP and IL-37 genes in groups of patients with active TB, healthy individuals with LTB and healthy controls without *M.tb* infection.

Our results for the first time showed a significant increase in the relative expression of IL-18 and IL-18BP mRNA in the group of patients with active TB and LTB individuals when compared to healthy controls, according to available literature data. It might suggest a permanent activation of the immune cell signaling pathways in the course of *M.tb* infection either in the control or progression to active TB disease. Moreover, no significant differences in relative IL-18 and IL-18BP mRNA expression were observed between active TB patients and LTB individuals. Pechkovsky et al. demonstrated an increased expression of IL-18 mRNA in type II lung epithelial cells obtained from patients with pulmonary TB. Pneumocytes that were cultured in the presence of *M.tb* cell lysate showed an increased IL-18 mRNA expression in comparison with unstimulated cells and pneumocytes stimulated with PPD or LPS [29]. Higher levels of IL-18 mRNA expression were observed in monocytes responding to *M. leprae* antigens [30]. Corbaz et al. showed significantly higher levels of IL-18 and IL-18BP mRNA expression in the intestinal mucosa of patients with Crohn’s disease as compared to healthy controls. We detected a similar increase in IL-18 and IL-18BP mRNA expression in the group of patients with active TB and healthy LTB individuals. These results are somewhat out of line with our earlier definition of a simultaneous increase in serum IL-18 and IL-18BP protein expression, which might be treated as a discriminatory biomarker of active tuberculosis and LTB. Yet, it is worth noting that only the complex co-expression of serum IL-18BP and IL-37, IP-10, and IFN-γ were identified as the most accurate discriminative biomarker set for diagnosis of active TB [21].

Our study allowed for the discovery of a novel relation—the significantly lower expression of functional IL-18R receptor mRNA in the LTBI group as compared to the active TB and healthy controls. This low expression of IL-18R mRNA in LTB individuals was accompanied by IFN-γ mRNA expression at the ’baseline’ level, characterizing healthy individuals without *M.tb* infection. Similarly, Taha et al. pointed out that the expression of IFN-γ genes was significantly higher in the group of patients with active TB as compared to those who were infected but did not develop active TB [31].

Among many possible mediators of host response to *M.tb* is the activity of the indoleamine 2,3-dioxygenase-1 (IDO1), the enzyme of tryptophan metabolism, which leads to the formation of tryptophan metabolites, including quinolinic and picolinic acids [32]. In animal models, increased IDO-1 expression and the activation of the tryptophan-kynurenine pathway were indicated to play a crucial role in *M.tb* pathogenesis [29,30,33,34]. The depletion of tryptophan, which is required for microbial growth, as well as the accumulation of biologically active tryptophan metabolites, impaired effective anti-mycobacterial immune response and, thus, favoured survival and persistence of the pathogen [32]. In *M.tb*-infected mice, IFN-γ receptor-deficiency in nonhaematopoietic cells led to a lack of IDO-1 expression and it was associated with exuberant neutrophil recruitment and increased mortality [33]. In macaques, the suppression of IDO activity led to the reduction of the bacterial burden and clinical symptoms of active TB that was accompanied by increased lung T cell proliferation, the induction of inducible bronchus-associated lymphoid tissue, and the relocation of effector T cells to the center of the granuloma [34]. Mehra et al. demonstrated that IDO induction in the periphery of the granuloma correlated with active TB disease [30]. A few studies have investigated IDO-mediated tryptophan metabolism and its metabolites in humans in the context TB. Li et al. demonstrated increased IDO expression and activity in the pleural fluid from TB patients [35]. Almeida et al. found significantly higher expression levels of immune-suppressive mediators, including IDO-1, in patients with active pulmonary TB as compared to patients with other infectious lung diseases and healthy volunteers [36]. The authors suggested that the increased levels of immunosuppressive mediators may render the immune activation and counteract the development of Th1-type immune response against *M.tb*. The IDO levels were elevated at the time of TB diagnosis and declined after TB treatment, which serves as evidence that IDO expression might be both: a useful diagnostic marker of active TB as well as prognostic factor in TB treatment of HIV-negative patients [36]. Additionally, Adu-Gyamfi et al. showed that plasma IDO expression is a potential biomarker of active TB in HIV-positive patients [37], while Shi et al. confirmed that IDO activity might have an auxiliary diagnosis value for the early discrimination of multi-drug resistant TB patients [38]. The increase in IDO activity was noticed in both HIV-infected and uninfected active TB patients as compared with individuals with latent TB infection [36,37,39]. In relation to this statement, the simultaneous reduction of IL-18R mRNA expression together with significant overexpression of IL-18 mRNA observed by us in the LTB group is of particular interest.

The presented results entitle us to hypothesize that: the increase in IL-18 gene expression, the lack of increase in IFN-γ gene expression, and the remarkably reduced expression of IL-18R gene may be a novel set of conditions that partially describe the homeostasis between *M.tb* and host-immunity in latent tuberculosis infection. As an obligate intracellular pathogen, *M.tb* has numerous adaptive mechanisms of modifying cellular processes in the fight against the host immune response. In latent TB infection, *M.tb* bacilli benefit from epigenetic changes that occurred in the host immune system under mycobacterial infection [40]. These changes make the *M.tb* favorable environment in the host cells and promote mycobacterial survival, growth, and latency. In a study that was conducted among Chinese patients with pulmonary TB and healthy controls, single nucleotide polymorphisms in the IL-18R promoter were associated with genotype-specific methylation status and genotype-specific IL-18R expression [41]. In the author’s opinion, the relationship between decreased mRNA expression of IL-18R that is caused by an SNP and increased DNA methylation can partially mediate the susceptibility to TB risk. No statistically significant differences were observed in the relative mRNA IL-37 expression among the groups in our study. IL-37 is a new member of the IL-1 family, which reduces systemic and local inflammation. IL-37 is expressed in various cells and tissues and it is regulated by numerous inflammatory stimuli and cytokines via different signal transduction pathways [42]. Mannose-capped lipoarabinomannan purified from *M.tb* induces IL-37 production via enhancing TLR2 expression in human type II alveolar epithelial cells; this process might contribute to the persistence of *M.tb* infection [43]. Zhao et al. indicated that IL-37 is also a negative regulator of immune responses in *Listeria monocytogenes* infection due to reduced production of colony-stimulating factors and increased macrophage apoptosis [44]. In our earlier studies, the serum concentration of the IL-37 protein was similar in the group of patients with active pulmonary TB and healthy individuals with or without latent *M.tb* infection [21]. However, the complex co-expression between the two IL-18 inhibitors, IL-18BP and IL-37, was identified as the strongest discriminative biomarker of active TB disease.

## 5. Conclusions

The role of IL-18, its binding protein IL-18BP, and IFN-γ in the development of the immune response against mycobacteria was confirmed by observing the increased level of the expression of these genes in the group of patients with active pulmonary TB. The reduced expression of IL-18R gene in healthy individuals with latent TB infection can, at least partially, prevent the development of a pathological inflammatory reaction and promote the maintenance of homeostatic conditions between host immunity and *M.tb* infection. In our future studies, we plan to test the expression of other genes that are tightly co-regulated with the IL-18 pathway.

## Figures and Tables

**Table 1:** Patient characteristics.

	ATB	LTB	Control
N	51	24	22
Sex M/F	40/11 *	8/16	5/17 *
Ethnicity	Caucasian	Caucasian	Caucasian
Age			
median	54	51	37
range	23–80	25–65	18–66
years (IQR)	42–63	45–57	27–42
BCG vaccination	100%	100%	100%
QFT result, N (%)			
positive	22 (43%)	24 (100%)	0 (0%)
negative	28 (57%)	0 (0%)	22 (100%)

Abbreviations: ATB—active tuberculosis patients; LTB—latently *M.tb* infected individuals; Control—*M.tb*-uninfected healthy controls; QFT—QuantiFERON TB Gold test.* The proportion of men in the ATB group was significantly higher than in the Control group (*p* < 0.05).

**Table 2:** Starters and temperatures of annealing selected for expression analysis.

	Sequence	Temperature of Annealing	Source
IL-18	forward 5’-GCTTGAATCTAAATTATCAGTC-3’	55 °C	[22]
	reverse 5’-GAAGATTCAAATTGCATCTTAT-3’		
IL-18BP	forward 5’-CAACTGGACACCAGACCTCA-3’	64 °C	[23]
	reverse 5’-AGCTCAGCGTTCCATTCAGT-3’		
IL-18R	forward 5’-GGACTCCATGAAGCATTGGT-3’	58 °C	[24]
	reverse 5’-AGACTCGGAAAGAACAGGCA-3’		
IFN-γ	forward 5’-CTCTTGGCTGTTACTGCCAGG-3’	60 °C	[25]
	reverse 5’-CTCCACACTCTTTTGGATGCT-3’		
IL-37	Sino Biological INC.	60 °C	-
